# Impact of Increasing Dietary Calcium Levels on Calcium Excretion and Vitamin D Metabolites in the Blood of Healthy Adult Cats

**DOI:** 10.1371/journal.pone.0149190

**Published:** 2016-02-12

**Authors:** Nadine Paßlack, Bettina Schmiedchen, Jens Raila, Florian J. Schweigert, Friederike Stumpff, Barbara Kohn, Konrad Neumann, Jürgen Zentek

**Affiliations:** 1 Institute of Animal Nutrition, Department of Veterinary Medicine, Freie Universität Berlin, Königin-Luise-Str. 49, 14195, Berlin, Germany; 2 Institute of Nutritional Science, University of Potsdam, Arthur-Scheunert-Allee 114–116, 14558, Bergholz-Rehbrücke, Germany; 3 Institute of Veterinary Physiology, Department of Veterinary Medicine, Freie Universität Berlin, Oertzenweg 19b, 14163, Berlin, Germany; 4 Clinic of Small Animals, Department of Veterinary Medicine, Freie Universität Berlin, Oertzenweg 19b, 14163, Berlin, Germany; 5 Institute of Biometry and Clinical Epidemiology, Charité –Universitätsmedizin Berlin, Hindenburgdamm 30, 12203, Berlin, Germany; University of Pittsburgh School of Medicine, UNITED STATES

## Abstract

**Background:**

Dietary calcium (Ca) concentrations might affect regulatory pathways within the Ca and vitamin D metabolism and consequently excretory mechanisms. Considering large variations in Ca concentrations of feline diets, the physiological impact on Ca homeostasis has not been evaluated to date. In the present study, diets with increasing concentrations of dicalcium phosphate were offered to ten healthy adult cats (Ca/phosphorus (P): 6.23/6.02, 7.77/7.56, 15.0/12.7, 19.0/17.3, 22.2/19.9, 24.3/21.6 g/kg dry matter). Each feeding period was divided into a 10-day adaptation and an 8-day sampling period in order to collect urine and faeces. On the last day of each feeding period, blood samples were taken.

**Results:**

Urinary Ca concentrations remained unaffected, but faecal Ca concentrations increased (*P* < 0.001) with increasing dietary Ca levels. No effect on whole and intact parathyroid hormone levels, fibroblast growth factor 23 and calcitriol concentrations in the blood of the cats were observed. However, the calcitriol precursors 25(OH)D_2_ and 25(OH)D_3_, which are considered the most useful indicators for the vitamin D status, decreased with higher dietary Ca levels (*P* = 0.013 and *P* = 0.033). Increasing dietary levels of dicalcium phosphate revealed an acidifying effect on urinary fasting pH (6.02) and postprandial pH (6.01) (*P* < 0.001), possibly mediated by an increase of urinary phosphorus (P) concentrations (*P* < 0.001).

**Conclusions:**

In conclusion, calcitriol precursors were linearly affected by increasing dietary Ca concentrations. The increase in faecal Ca excretion indicates that Ca homeostasis of cats is mainly regulated in the intestine and not by the kidneys. Long-term studies should investigate the physiological relevance of the acidifying effect observed when feeding diets high in Ca and P.

## Introduction

Calcium (Ca) homeostasis in cats is hormonally regulated by calcitriol, parathyroid hormone (PTH) and calcitonin [1]. Both, PTH and calcitriol enhance Ca and phosphate mobilisation from bones. In addition, PTH particularly increases Ca and inhibits phosphate reabsorption in the tubules of the kidneys [1]. Calcitriol further enhances intestinal Ca and phosphate absorption, while calcitonin lowers blood Ca concentration by Ca accretion in bones [1].

In addition to these hormones, fibroblast growth factor 23 (FGF23) has also been recognized to affect Ca and phosphorus (P) metabolism [2]. FGF23 increases renal P excretion, decreases gastrointestinal P absorption, downregulates PTH and decreases plasma calcitriol concentrations [2]. In this way, not only calcitriol, but also plasma P concentrations are reduced by FGF23 [2]. It has been demonstrated that FGF23 is elevated in cats with chronic kidney disease, and increases with disease progression [3]. In particular, FGF23 plasma concentrations are elevated in cats with CKD and associated azotemia and hyperphosphatemia [3]. Another study found increased FGF23 plasma concentrations in cats even before the development of azotemia when compared to cats that did not develop azotemia afterwards, indicating that FGF23 might be considered as a potential biomarker for disorders in P metabolism in cats in the future [4].

Besides the effect of blood phosphate levels on FGF23 secretion, Ca concentrations in the blood might also be relevant for FGF23 regulation [2]. Rats fed a low Ca diet showed hypocalcemia and associated decreased plasma FGF23 concentrations [5], while feeding a high Ca diet to mice increased both, plasma Ca and FGF23 concentrations [6]. However, data in humans suggest that only long-term hypercalcemia might affect FGF23 secretion, since an acute increase of blood Ca levels was not accompanied by an increase in FGF23 concentrations [7].

To date, no studies have been conducted in order to evaluate the impact of varying dietary Ca levels on hormonal balance in cats, particularly with regard to vitamin D metabolism. Studies in rats have demonstrated that Ca deprivation lowers 25(OH)D levels in the blood [8, 9], which might promote vitamin D deficiency and associated disorders [8]. Moreover, a higher Ca intake was associated with longer plasma half life of 25(OH)D [8], why a vitamin D sparing effect by high Ca intakes has been assumed [10].

The recommended allowance for dietary Ca in healthy adult cats is indicated with 2.9 g/kg dry matter (DM), assuming an energy density of 16.7 MJ metabolizable energy (ME)/kg [11]. However, large variations in feline Ca intake can be supposed. On the one hand, commercial diets might differ from the recommendations. The European Pet Food Industry Federation (FEDIAF) [12] recommends a minimum dietary Ca concentration of 10.0 g/kg DM, and the Association of American Feed Control Officials (AAFCO) [13] indicates an adult maintenance minimum of 6.0 g Ca/kg DM for cats. On the other hand, home-prepared diets for companion animals can show nutritional imbalances and variations in Ca concentration [14, 15].

To date, the physiological significance of varying Ca intakes in cats has scarcely been evaluated. It can be supposed that hormonal and excretory mechanisms might be triggered by high or low dietary Ca concentrations. In addition, the metabolic effects may not exclusively depend on the dietary level, but also on the source of Ca and the Ca:phosphorus (P)-ratio. Related changes in mineral metabolism might especially affect acid base balance [16–20] or urine composition [21, 22].

In our previous study, higher dietary Ca levels were accompanied by reduced parathyroid hormone (PTH) concentrations in the blood of cats [23], indicating a dietary regulation of calciotropic hormones. The present study aimed at investigating these hormonal mechanisms in the feline organism in more detail, particularly focussing on the interaction between dietary Ca level and vitamin D metabolites in the blood. Vitamin D metabolites have only infrequently measured in cats, and to our best knowledge, no interactions between dietary Ca levels and vitamin D metabolites in cats have been evaluated to date. Moreover, concentrations of FGF23, an important factor for P homeostasis, were determined in the blood of the cats. Therefore, the data of the present study will provide new information on regulatory mechanisms within the feline Ca and P metabolism depending on dietary Ca and P supply.

## Material and Methods

### Experimental design

The experimental protocol was approved by the Animal Welfare Committee (Landesamt für Gesundheit und Soziales, Berlin, Germany, G 0024/11). Ten healthy adult cats (European Shorthair, 5 neutered males, 5 intact females, 47 ± 17 months) of the cat colony of the Institute of Animal Nutrition, Freie Universität Berlin, received 6 experimental diets in 6 feeding periods. All cats were fed the same diet at the same time. The sequential study design was considered based on the findings of Clements et al. [8] that half life of 25(OH)D was linearly enhanced by increasing dietary Ca concentrations, indicating a Ca-dependent sparing effect on blood vitamin D [10]. To exclude possible cross-over-effects of the respective preceding diet on the subsequent feeding period, the diets were fed in ascending order, starting with the diet with the lowest Ca concentration. Thus, no significant impact of the respective previous feeding period on the measurements can be assumed. Since environmental conditions were kept constant during the study period, direct effects of the time or the duration of the study on the results are not to be expected. Regarding the total study duration, age effects of the cats can be excluded. Each feeding period was divided into a 10-day adaptation period and an 8-day sampling period to collect the urine and faeces of the cats. On the last day of each feeding period, one blood sample per cat was taken.

### Animal housing and method of sampling

The cats were housed as a group (adaptation period) or separately in metabolic cages (sampling period) in a room under constant light (12 h light—12 h darkness) and temperature (21°C) conditions. They were fed individually twice a day at 07.00 and 12.00 hours, and the daily feed intake was documented for each cat throughout the study. Purified water was offered ad libitum. The body weight (BW) of the cats was measured once a week.

The metabolic cages contained purpose-built cat litter boxes with plastic pellets as litter and adapted urine collection containers. The urine could directly flow into these containers, and the faeces of the cats remained in the litter boxes. The urine collection containers were provided with three drops of chlorhexidine-digluconate to prevent bacterial growth in the urine. The total urine of the cats was collected twice a day during the sampling periods. After measuring the urinary pH, the samples were stored at -40°C until further analysis. The total faecal samples of the cats were also collected twice a day during the sampling periods and stored at -40°C. The blood of the cats was collected in the morning of the last day of each feeding period, when the animals were fasting for at least 18 hours.

### Diets

Six experimental diets were offered in six feeding periods. The Ca and P levels were 6.23/6.02, 7.77/7.56, 15.0/12.7, 19.0/17.3, 22.2/19.9 and 24.3/21.6 g/kg dry matter (DM), respectively (Table 1). Considering dietary Ca concentrations which can usually be found in commercially available cat food, the Ca levels in the experimental diets therefore represent low-Ca (diets 0.6% and 0.8% Ca) normal and high-Ca (diets 1.9–2.4% Ca) diets. Dicalcium phosphate (CaHPO_4_) was used as dietary Ca source. The diets were formulated to fulfil the recommendations for adult cats [11]. For all experimental diets, the same premix with trace elements and vitamins was used, ensuring similar concentrations of these nutrients among the diets. The premix contained vitamin D_3_ as source of vitamin D. The nutrient analysis of the experimental diets was performed as described elsewhere [24].

**Table 1:** Nutrient analysis of the experimental diets^1^.

		Group (% Ca in DM)					
Analysed composition		0.6	0.8	1.5	1.9	2.2	2.4
Dry matter	g/kg	904	917	926	931	921	959
Crude protein	g/kg DM	339	352	354	344	342	329
Crude fat	g/kg DM	174	166	122	158	173	122
Crude fiber	g/kg DM	12.9	15.1	7.11	7.30	5.73	13.7
Crude ash	g/kg DM	44.8	49.1	73.0	87.4	98.4	107
Calcium	g/kg DM	6.23	7.77	15.0	19.0	22.2	24.3
Phosphorus	g/kg DM	6.02	7.56	12.7	17.3	19.9	21.6
Calcium:Phosphorus-ratio		1.03:1	1.03:1	1.18:1	1.10:1	1.12:1	1.13:1
Sodium	g/kg DM	4.08	3.88	3.75	3.69	3.53	3.52
Potassium	g/kg DM	6.57	5.78	6.00	6.24	5.97	5.62
Magnesium	g/kg DM	0.85	0.84	0.76	0.74	0.70	0.86
Chloride	g/kg DM	8.45	8.48	8.41	8.43	9.09	6.50
Methionine	g/kg DM	7.34	7.64	8.01	8.21	7.71	6.77
Cysteine	g/kg DM	9.40	9.76	9.82	9.38	9.47	9.29
Metabolizable energy^2^	MJ/kg DM	18.8	18.5	17.3	17.8	18.0	16.5
Base excess^3^	mmol/kg DM	-155	-218	-200	-297	-332	-246

^1^Ingredients list: corn (whole grain), wheat (whole grain), dried greaves, corn gluten, animal fat, poultry meal, dried beet pulp, dicalcium phosphate, other minerals, vitamins and digest (hydrolysed meat, dried); The vitamin D_3_ concentration was 1650 IU/kg diet (according to the manufacturer)

^2^Calculated according to NRC [11]

^3^Calculated as follows [20]: Base excess (mmol/kg DM) = 49.9*Ca (g/kg DM) + 82.3*magnesium (Mg) (g/kg DM) + 43.5*sodium (Na) (g/kg DM) + 25.6*potassium (K) (g/kg DM) - 64.6*P (g/kg DM) - 13.4*methionine (g/kg DM) - 16.6*cysteine (g/kg DM) - 28.2*ch1oride (Cl) (mg/kg DM).

The base excess (BE) was calculated as follows [20]:

BE (mmol/kg DM) = 49.9*Ca (g/kg DM) + 82.3*magnesium (Mg) (g/kg DM) + 43.5*sodium (Na) (g/kg DM) + 25.6*potassium (K) (g/kg DM) - 64.6*P (g/kg DM) - 13.4*methionine (g/kg DM) - 16.6*cysteine (g/kg DM) - 28.2*ch1oride (Cl) (mg/kg DM)

Based on the BE in the diet, the urinary pH was calculated [20]:

Urinary pH = (BE*0.0021) + 6.72

### Blood analysis

The blood was collected in serum and EDTA tubes and stored at room temperature for one hour. The EDTA tubes were placed in the fridge at 4°C, and the serum tubes were centrifuged at 4°C and 1811 x g for 10 min. (Heraeus Megafuge 1.0R, Thermo Scientific, Karlsruhe, Germany). The mineral concentrations in the serum were measured photometrically (Konelab 60 i Thermo Fisher Scientific, Passau, Germany). The blood count as well as the measurement of urea and creatinine was performed using automatic methods (Sysmex XT-2000i Sysmex GmbH, Norderstedt, Germany; Konelab 60 i Thermo Fisher Scientific).

For the quantitative measurement of whole and intact PTH (wPTH and iPTH), the commercial Duo PTH Kit (Scantibodies Laboratory, Inc., Santee, CA, USA; Part Number: 3KG601) was used, which has been validated for cats [25]. The concentrations of wPTH and iPTH in the serum of the cats were determined according to the instructions of the manufacturer.

FGF23 concentrations in the serum of the cats were measured using a commercial ELISA kit (Kainos Laboratories, Inc., Tokyo, Japan). The test was previously validated in cats [3]. Sample preparation and FGF23 measurements were performed as specified by the manufacturer.

The vitamin D_2_ and D_3_ metabolites in the serum of the cats were assayed by LC/MS-MS (Agilent, Böblingen, Germany) using a modified method described by Aronov et al. [26]. In brief, 250 μL serum samples were spiked with internal hexadeuterated vitamin D standards and deproteinized with acetonitrile. The samples were then extracted twice using methyl tert-butyl ether. The organic phases were pooled and evaporated to dryness and reconstituted with 25 μL of the derivatization agent 4-Phenyl-1,2,4-triazoline-3,5-dione. After 1 h incubation at room temperature, the samples were separated using an Agilent Zorbax SB C18 column on an Agilent 1260 HPLC system. Mass spectrum analysis was carried out using the positive mode electrospray ionization method on an Agilent 6460 triple quadrupole tandem mass spectrometer. Multiple reaction monitoring channels were set to identify vitamin D analytes. The analysis of vitamin D_2_ and vitamin D_3_ was optimized using standards solved in acetonitrile (20 ng/mL; Sigma-Aldrich, Munich, Germany). The linear range covered by these metabolites was 0.02–25 ng/mL, R^2^ = 0.984. Lower limit of quantification was 0.05 ng/ml. The intra-assay and inter-assay coefficients of variance for this analysis were < 15%.

### pH measurements and preparation of the urine samples

The urinary pH of the cats was measured at 07.00 hours (fasting pH) and 12.00 hours (postprandial pH). The measurements were performed with an electronic pH meter (Seven Multi, Mettler-Toledo GmbH, Schwerzenbach, Switzerland).

For the analysis of urine composition, the urine was defrosted at room temperature. After measuring the total urine volume of each cat, a sample of 12 ml was taken for further analysis and the remainder frozen at -80°C. The pH of the sample was adjusted to a pH of 2 using hydrochloric acid (37%) (pH-meter Seven Multi, Mettler-Toledo). The added amount of hydrochloric acid was documented. The urine was filtered (Syringe Filter, Bulk, SFCA (surfactant-free cellulose acetate), 0,2μm, 25mm non-sterile, Thermo Scientific, Rochester, NY, USA) prior to the further analysis [23].

### Preparation of the faecal samples

The frozen faecal samples were weighed and lyophilized in a vacuum freeze-dryer (Lyovac GT2, LC Didactic, Hürth, Germany) for a duration of 3 days at the minimum or until weight constancy. In order to separate the plastic pellets of the cat litter boxes, the plastic pellets were removed manually before diminution (Mill: ZM 100, Retsch, Haan, Germany). The faeces were ground to a particle size of 0.25 mm and stored in glass boxes prior to the following mineral analysis [23].

### Statistical analysis

The data were analysed using R version 3.0 (http://www.r-project.org/). For each subject (cat), a quadratic dependence of all variables on the dietary Ca concentration was assumed. These individual models were combined into a mixed effects model with intercept, linear and quadratic effect as fixed and random variables (two stage model; c.f. [27]). *P*-values for the linear and quadratic component of the orthogonal decomposition of the effect were computed by the function lme from the R package nlme 3.1. A mixed model was used instead of repeated measure analysis since two cats refused the diet with the highest Ca concentration. The data were presented in tables as means and standard deviations. The level of significance was *P* < 0.05. Since the statistical analysis is exploratory, no Bonferroni adjustment of the level of significance was performed.

## Results

### Animal health, BW and feed intake

All cats were healthy throughout the study and no effect of the diets on the BW of the animals was observed. The daily feed intake did not differ among the groups, however, two cats refused the diet 2.4% Ca. Therefore, the number of animals was n = 8 for all measured parameters in this feeding group.

### Blood

**Fig 1:**
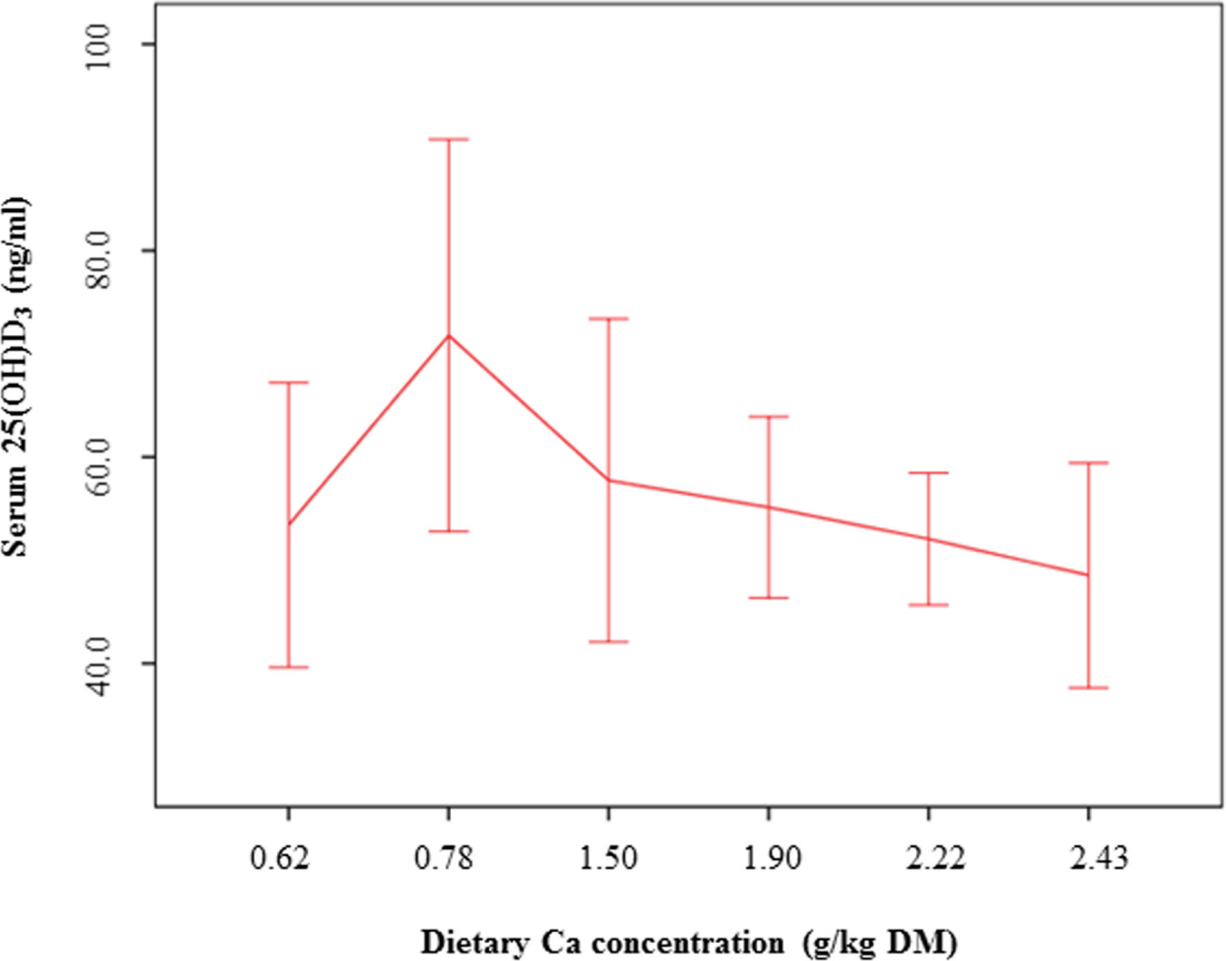
Serum 25(OH)D_3_ concentrations of cats fed a diet with different levels of dicalcium phosphate. Means and 95% confidence intervals.

**Fig 2:**
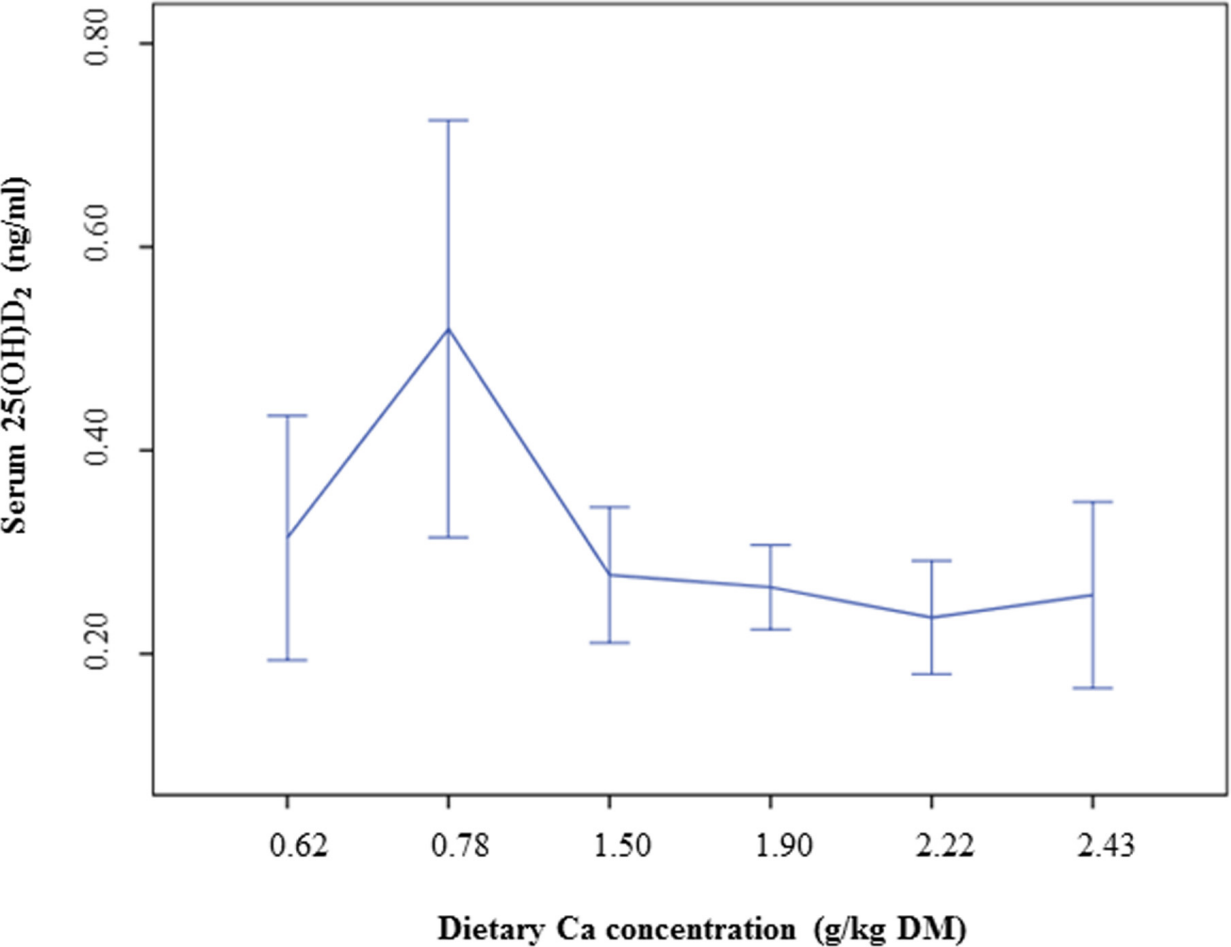
Serum 25(OH)D_2_ concentrations of cats fed a diet with different levels of dicalcium phosphate. Means and 95% confidence intervals.

Vitamin D3 as well as the vitamin D metabolites 1α,25(OH)_2_D_3_ and 24,25(OH)_2_D_3_ were not affected by increasing dietary Ca levels. We found a significant decrease of 25(OH)D_2_ and 25(OH)D_3_ (linear contrast: *P* = 0.013 and *P* = 0.033, respectively) (Table 2), however, as shown in Figs 1 and 2, 25(OH)D_2_ and 25(OH)D_3_ concentrations in the blood of the cats were the highest at the low Ca diet “0.78% Ca”.

**Table 2:** Urea, creatinine, minerals, PTH, FGF23 and vitamin D metabolites in the blood of cats fed a diet with different levels of dicalcium phosphate. Mean and standard deviation, n = 10 / diet^1^

	Group (% Ca in DM)						Polynomial contrasts *(P*-values)		Reference range^2^
	0.6	0.8	1.5	1.9	2.2	2.4	Lin.	Quadr.	
Urea (mg/dl)	49.6 ± 5.30	44.4 ± 5.14	51.5 ± 9.55	56.9 ± 7.81	39.2 ± 5.24	43.9 ± 5.78	0.071	**0.034**	30.0–68.0
Creatinine (mg/dl)	1.73 ± 0.20	1.50 ± 0.20	1.52 ± 0.21	1.49 ± 0.21	1.54 ± 0.14	1.55 ± 0.24	**0.034**	**0.001**	0.60–1.80
Ca (mmol/l)	2.51 ± 0.11	2.41 ± 0.10	2.39 ± 0.09	2.39 ± 0.10	2.46 ± 0.09	2.53 ± 0.13	0.328	**< 0.001**	2.30–3.00
Phosphate (mmol/l)	1.35 ± 0.16	1.29 ± 0.14	1.37 ± 0.18	1.33 ± 0.22	1.35 ± 0.16	1.39 ± 0.20	0.313	0.393	0.80–1.90
Whole PTH (pg/ml)	7.25 ± 5.14	9.70 ± 7.08	9.77 ± 5.56	5.79 ± 4.04	9.34 ± 8.27	10.3 ± 5.74	0.488	0.575	
Intact PTH (pg/ml)	10.4 ± 7.13	9.76 ± 6.24	12.5 ± 5.05	4.38 ± 2.46	7.17 ± 5.97	11.7 ± 7.30	0.188	0.242	
FGF23 (pg/ml)	101 ± 58.0	110 ± 109	112 ± 55.2	105 ± 44.7	109 ± 42.9	134 ± 53.9	0.452	0.693	
1α,25(OH)_2_D_3_ (ng/ml)	0.56 ± 0.59	0.74 ± 0.35	0.59 ± 0.19	0.67 ± 0.22	0.59 ± 0.15	0.49 ± 0.15	0.628	0.341	
24,25(OH)_2_D_3_ (ng/ml)	27.4 ± 35.1	25.9 ± 12.8	18.8 ± 9.84	15.2 ± 5.83	16.8 ± 6.67	13.3 ± 7.54	0.122	0.685	
25(OH)D_2_ (ng/ml)	0.31 ± 0.17	0.52 ± 0.29	0.28 ± 0.09	0.27 ± 0.06	0.24 ± 0.08	0.26 ± 0.11	**0.013**	0.780	
25(OH)D_3_ (ng/ml)	53.4 ± 19.3	71.8 ± 26.5	57.7 ± 21.9	55.1 ± 12.3	52.1 ± 8.95	48.5 ± 13.0	**0.033**	0.063	
Vitamin D_3_ (ng/ml)	2.86 ± 3.13	7.08 ± 6.64	1.26 ± 0.59	7.13 ± 5.23	2.13 ± 0.93	0.50 ± 0.30	0.101	0.069	

^1^For the group 3.0% Ca: n = 8

^2^Clinic of Small Animals, Freie Universität Berlin.

The concentrations of wPTH, iPTH and FGF23 in the serum of the cats were not affected by the varying Ca concentrations in the diets (*P* > 0.05).

The experimental diets did not affect the hematological parameters of the cats (data not shown). Although some variations were observed for the urea (quadratic contrast: *P* = 0.034) and creatinine (linear contrast: *P* = 0.034; quadratic contrast: *P* = 0.001) concentrations among the experimental groups, all blood values were within the normal range for cats. The same applied for the Ca concentrations in the serum of the cats, which varied between 2.39–2.53 mmol/l (quadratic contrast: *P* < 0.001). The serum phosphate concentrations were not affected by the experimental diets (*P* > 0.05).

### Urine

Table 3 summarizes the data on the measured urine volume, the urinary pH and the urinary mineral concentrations among the feeding groups. The urine volume increased up to a dietary Ca concentration of 1.9% and subsequently decreased in the groups 2.2–2.4% Ca (quadratic contrast: *P* = 0.002).

**Table 3:** Body weight (BW), daily feed intake, urine volume and urinary mineral concentrations of cats fed a diet with different levels of dicalcium phosphate. Mean and standard deviation, n = 10 / diet^1^.

	Group (% Ca in DM)						Polynomial contrasts *(P*-values)	
	0.6	0.8	1.5	1.9	2.2	2.4	Lin.	Quadr.
ᴓ BW (kg)	4.13 ± 1.32	4.00 ± 1.28	4.07 ± 1.29	4.10 ± 1.33	4.17 ± 1.31	4.11 ± 1.45	0.140	0.515
Feed intake (g DM/kg BW/d)	12.7 ± 2.39	13.1 ± 2.32	13.1 ± 3.07	13.5 ± 2.37	13.2 ± 2.45	12.7 ± 3.99	0.798	0.137
Urine volume (ml/kg BW/d)	6.38 ± 2.77	10.2 ± 3.55	9.44 ± 4.02	9.99 ± 2.83	9.51 ± 3.43	8.49 ± 2.52	0.216	**0.002**
Calculated urinary pH^2^	6.39			6.26			6.30			6.10			6.02			6.20				
**Urinary**								
Fasting pH	7.09 ± 0.29	6.52 ± 0.17	6.29 ± 0.29	6.10 ± 0.23	6.02 ± 0.08	6.22 ± 0.08	**< 0.001**	**< 0.001**
Postprandial pH	6.91 ± 0.23	6.68 ± 0.23	6.41 ± 0.51	6.17 ± 0.32	6.01 ± 0.22	6.19 ± 0.29	**< 0.001**	**< 0.001**
Ca (mg/l)	26.2 ± 21.9	19.1 ± 5.48	27.3 ± 9.64	25.8 ± 9.24	25.1 ± 8.28	24.6 ± 7.60	0.808	0.982
P (mg/l)	2321 ± 328	3075 ± 588	5638 ± 959	6316 ± 879	7111 ± 980	7206 ± 994	**< 0.001**	**< 0.001**
Sulphate (mg/l)	4009 ± 885	4117 ± 869	4585 ± 904	4457 ± 679	4191 ± 681	4215 ± 629	0.463	**0.004**
Ox (mg/l)	174 ± 32.7	151 ± 32.3	167 ± 40.0	138 ± 30.6	123 ± 15.6	126 ± 13.0	**< 0.001**	0.931
Citrate (mg/l)	133 ± 115	84.2 ± 52.9	41.5 ± 41.9	31.4 ± 26.4	38.5 ± 30.1	111 ± 83.6	**0.006**	**0.001**
**Renal excretion**								
Ca (mg/kg BW/d)	0.13 ± 0.08	0.19 ± 0.05	0.25 ± 0.14	0.25 ± 0.10	0.23 ± 0.10	0.21 ± 0.09	0.052	**0.019**
P (mg/kg BW/d)	14.6 ± 6.68	30.0 ± 6.18	52.3 ± 20.9	62.5 ± 17.4	67.2 ± 25.7	61.3 ± 20.3	**< 0.001**	**< 0.001**

^1^For the group 3.0% Ca: n = 8

^2^ Calculated based on the base excess (BE) in the diet [20]: urinary pH = (BE*0.0021) + 6.72.

The fasting and postprandial urinary pH decreased with increasing levels of Ca in the diets, reaching values from 7.09/6.91 to 6.02/6.01 (diets 0.6–2.2% Ca; linear contrast: *P* < 0.001 for the fasting and postprandial urinary pH, respectively). However, the urinary pH marginally increased up to 6.22/6.19 after feeding the diet 2.4% Ca (quadratic contrast: *P* < 0.001 for the fasting and postprandial urinary pH, respectively). Considering the calculated urinary pH based on the BE in the diets, a decrease with increasing dietary Ca levels was also observed. The calculated pH values were similar with the measured urinary pH (diets 1.5–2.4% Ca) or approximately 5–10% lower (diets 0.6–0.8% Ca).

**Fig 3:**
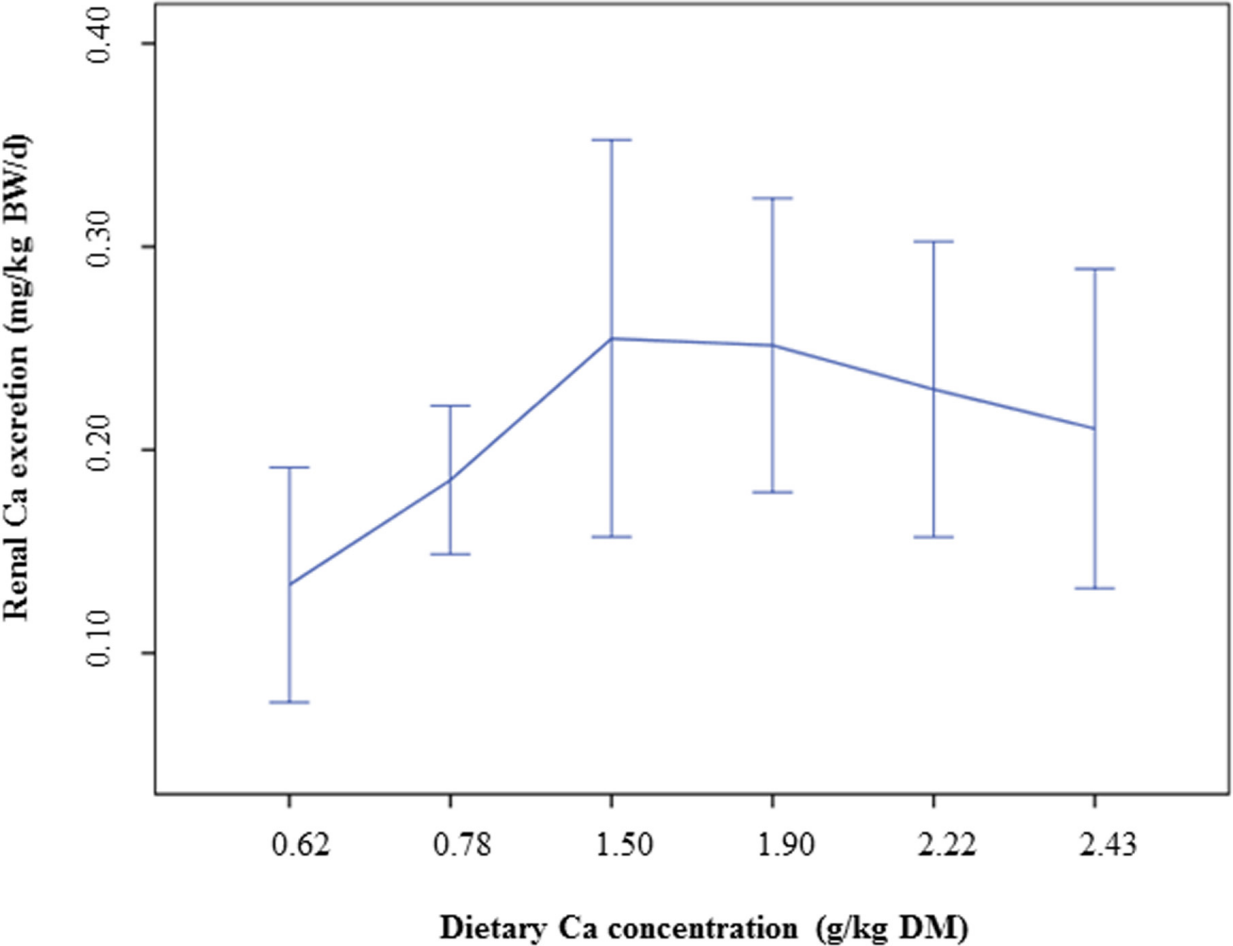
Renal Ca excretion of cats fed a diet with different levels of dicalcium phosphate. Means and 95% confidence intervals.

The urinary Ca concentrations were not affected by varying Ca levels in the diets (*P* > 0.05). The renal Ca excretion was small and decreased at low dietary Ca levels (0.6% Ca and 0.8% Ca; Fig 3) (quadratic contrast: *P* = 0.019).

**Fig 4:**
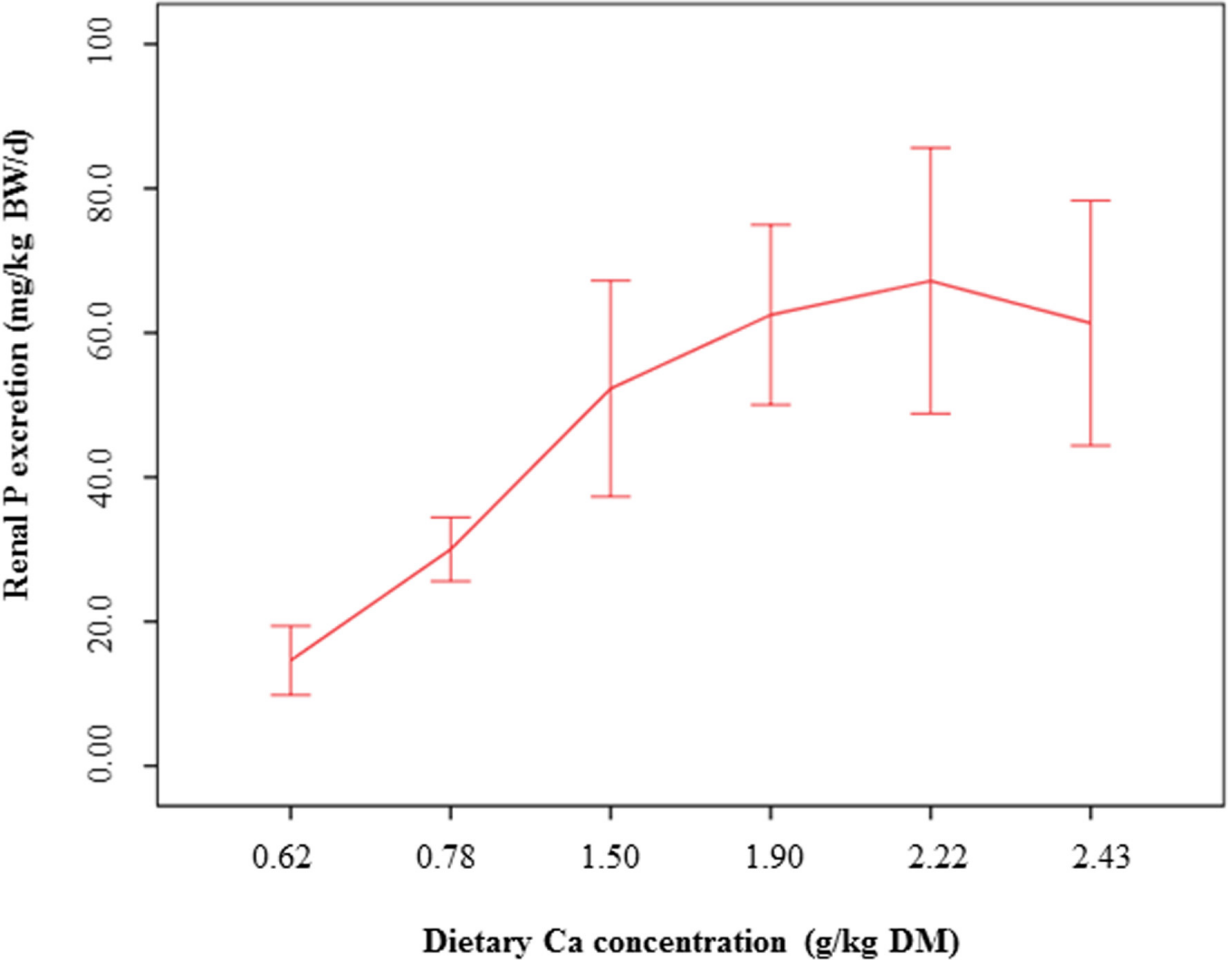
Renal P excretion of cats fed a diet with different levels of dicalcium phosphate. Means and 95% confidence intervals.

With increasing dietary dicalcium phosphate levels, urinary P concentrations increased more than threefold from 2321 mg/l to 7206 mg/l (linear contrast: *P* < 0.001). However, after a sharp increase for lower dietary P levels, the urinary P concentrations flattened out for higher P levels in the diets (quadratic contrast: *P* < 0.001). As demonstrated in Fig 4, the renal P excretion showed a sharp increase from 14.6 mg/kg BW/d (0.6% Ca) to 67.2 mg/kg BW/d (2.2% Ca), followed by a decrease down to 61.3 mg/kg BW/d (2.4% Ca) (*P* < 0.001 for linear and quadratic contrast, respectively).

The urinary oxalate (Ox) concentrations decreased from 174 mg/l to 126 mg/l with increasing dietary Ca levels (*P* < 0.001). The citrate concentrations first decreased from 133 mg/l (0.6% Ca) to 31.4 mg/l (1.9% Ca), but then increased up to 111 mg/l (2.4% Ca) (linear contrast: *P* = 0.006; quadratic contrast: *P* = 0.001).

A quadratic effect was observed for the urinary sulphate concentrations (*P* = 0.004).

### Faeces

The faecal DM content increased from 35.0% (0.6–0.8% Ca) to 45.8% (2.2% Ca) (linear contrast: *P* < 0.001) and moderately decreased to 43.6% after feeding the diet 2.4% Ca (quadratic contrast: *P* = 0.017) (Table 4). The daily amount of faeces showed an increase from 8.00 g DM to 12.9 g DM with the higher levels of Ca in the diets (linear contrast: *P* < 0.001).

The Ca concentrations in the faeces markedly increased with increasing dietary Ca levels from 36.3 mg/g DM (on the 0.6% Ca diet) to 107–120 mg/g DM (1.9–2.4% Ca diet) (linear contrast: *P* < 0.001). The faecal Ca excretion increased from 73.0 mg/kg BW/d (0.6% Ca) to 416 mg/kg BW/d (2.4% Ca) (linear contrast: *P* < 0.001), while the apparent digestibility of Ca was not affected by the diets (*P* > 0.05).

**Table 4:** Faecal mineral excretion of cats fed a diet with different levels of dicalcium phosphate. Mean and standard deviation, n = 10 / diet^1^.

	Group (% Ca in DM)						Polynomial contrasts *(P*-values)	
	0.6	0.8	1.5	1.9	2.2	2.4	Lin.	Quadr.
Amount of faeces (g/d)	23.0 ± 7.22	22.6 ± 7.57	23.7 ± 10.2	23.7 ± 7.18	21.1 ± 7.84	30.6 ± 12.4	0.152	0.069
DM of the faeces (%)	35.0 ± 3.70	35.0 ± 2.72	42.3 ± 4.30	42.3 ± 3.63	45.8 ± 5.56	43.6 ± 6.07	**< 0.001**	**0.017**
Amount of faeces (g DM/d)	8.00 ± 2.39	7.80 ± 2.26	9.73 ± 3.55	9.88 ± 2.68	9.39 ± 2.71	12.9 ± 4.43	**< 0.001**	0.374
Faecal Ca (mg/g DM)	36.3 ± 3.26	50.9 ± 3.50	83.2 ± 12.4	107 ± 7.45	119 ± 11.0	119 ± 30.7	**< 0.001**	**0.006**
Faecal Ca excretion (mg/kg BW/d)	73.0 ± 18.4	101 ± 18.0	208 ± 89.5	266 ± 56.6	276 ± 58.0	416 ± 251	**< 0.001**	0.868
Apparent digestibility of Ca (%)	8.49 ± 10.2	- 0.44 ± 16.5	- 4.24 ± 32.1	- 4.54 ± 17.5	4.36 ± 19.6	- 28.7 ± 41.2	0.102	0.653
Faecal P (mg/g DM)	23.8 ± 1.86	28.7 ± 3.21	50.8 ± 3.43	62.2 ± 2.30	72.0 ± 3.72	60.1 ± 11.7	**< 0.001**	**< 0.001**
Faecal P excretion (mg/kg BW/d)	48.8 ± 16.5	57.1 ± 11.7	125 ± 45.1	155 ± 29.5	166 ± 28.9	193 ± 56.7	**< 0.001**	0.052
Apparent digestibility of P (%)	36.8 ± 15.5	41.7 ± 11.3	25.6 ± 16.9	33.4 ± 10.5	36.1 ± 8.72	28.1 ± 15.8	0.288	0.720

^1^For the group 3.0% Ca: n = 8.

The faecal P concentrations increased from 23.8 mg/g DM after feeding the diet 0.6% Ca up to 72.0 mg/g DM, when the diet 2.2% Ca was fed (linear contrast: *P* < 0.001). However, a moderate decrease to 60.1 mg P/g DM was observed when the cats received the diet 2.4% Ca (quadratic contrast: *P* < 0.001). The faecal P excretion showed a strict increase with increasing levels of dicalcium phosphate in the diets (linear contrast: *P* < 0.001), and the apparent digestibility of P was not affected by the experimental diets (*P* > 0.05).

## Discussion

Daily Ca intake might markedly differ between cats, considering variations in feeding regimens and composition of feline diets. Previous studies in rats have demonstrated reduced 25(OH)D and higher 1,25D concentrations in the blood of the animals at dietary Ca deprivation when compared to higher dietary Ca levels [8, 9]. In the present study, no group differences were observed for calcitriol (1α,25(OH)_2_D_3_), the hormonally active form of vitamin D. However, the significant decrease of the precursors of calcitriol, 25(OH)D_2_ and 25(OH)D_3_, associated with increasing dietary Ca concentrations, indicate a counterregulative effect caused by the dietary Ca level. The level of 25(OH)D_3_ is considered to reflect the vitamin D status of the organism [28]. It is the main circulating form of vitamin D [1, 29] and the concentrations in the blood are more stable than those of calcitriol or vitamin D [30]. The present data on the serum 25(OH)D_2_ and 25(OH)D_3_ concentrations can therefore be assumed to be reliable indicators for the vitamin D status of the cats depending on the dietary Ca levels. However, the data contrast with the results observed in rats [8, 9], since higher, but not reduced, serum 25(OH)D concentrations were associated with low dietary Ca levels. It should be considered that the present study has not investigated Ca deprivation in cats, but generally focussed on variations in dietary Ca intake and the impact on regulatory mechanisms in the feline organism. Ca and vitamin D requirements [11] were fulfilled by the experimental diets, but the 0.5% Ca diet was below the recommended minimum dietary Ca concentration indicated by the FEDIAF [12]. In the studies of Clements et al. [8] and Anderson et al. [9], Ca deprivation was induced in rats for 6 months. Thus, differences between the results obtained in rats and in the cats of the present study might be explained by differences in the study design, especially with regard to achieved dietary Ca concentrations and duration of the feeding periods. However, species-related differences in vitamin D metabolism cannot be excluded when the present results are compared with data obtained in rats [8, 9] and should be investigated further in future studies.

**Fig 5:**
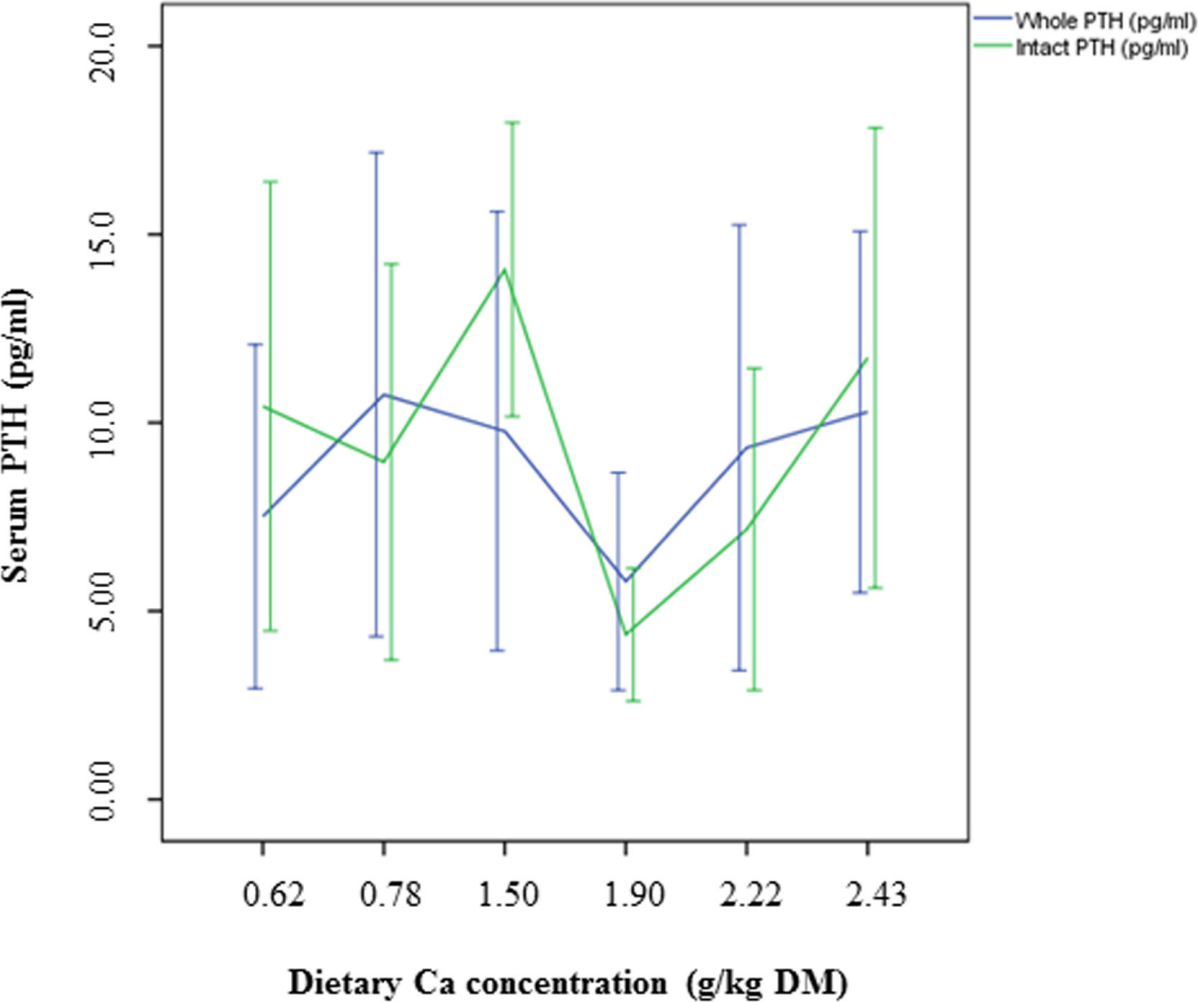
Serum PTH concentrations of cats fed a diet with different levels of dicalcium phosphate.Means and 95% confidence intervals.

**Fig 6:**
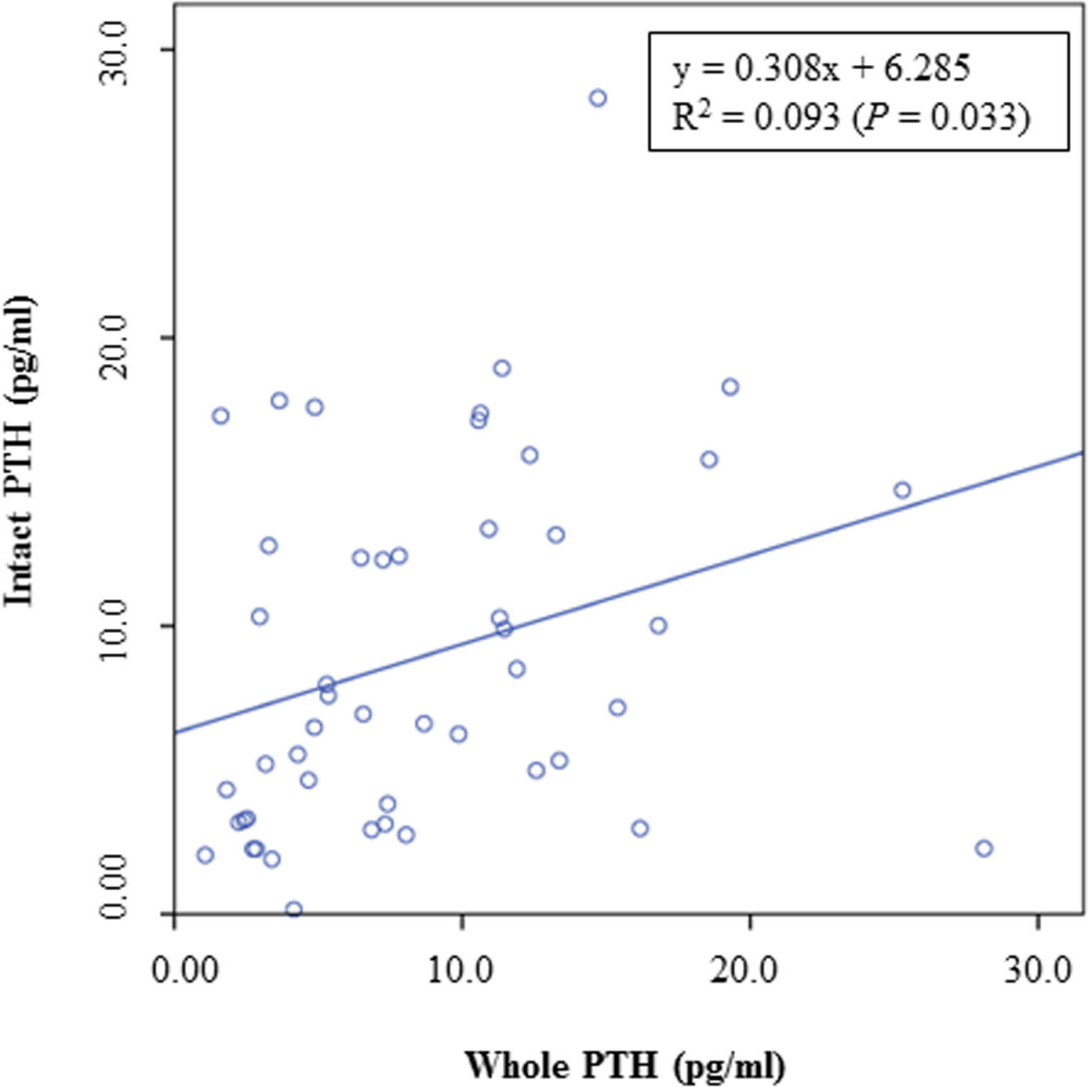
Concentrations of iPTH as a function of wPTH in the serum of cats fed a diet with different levels of dicalcium phosphate.

**Fig 7:**
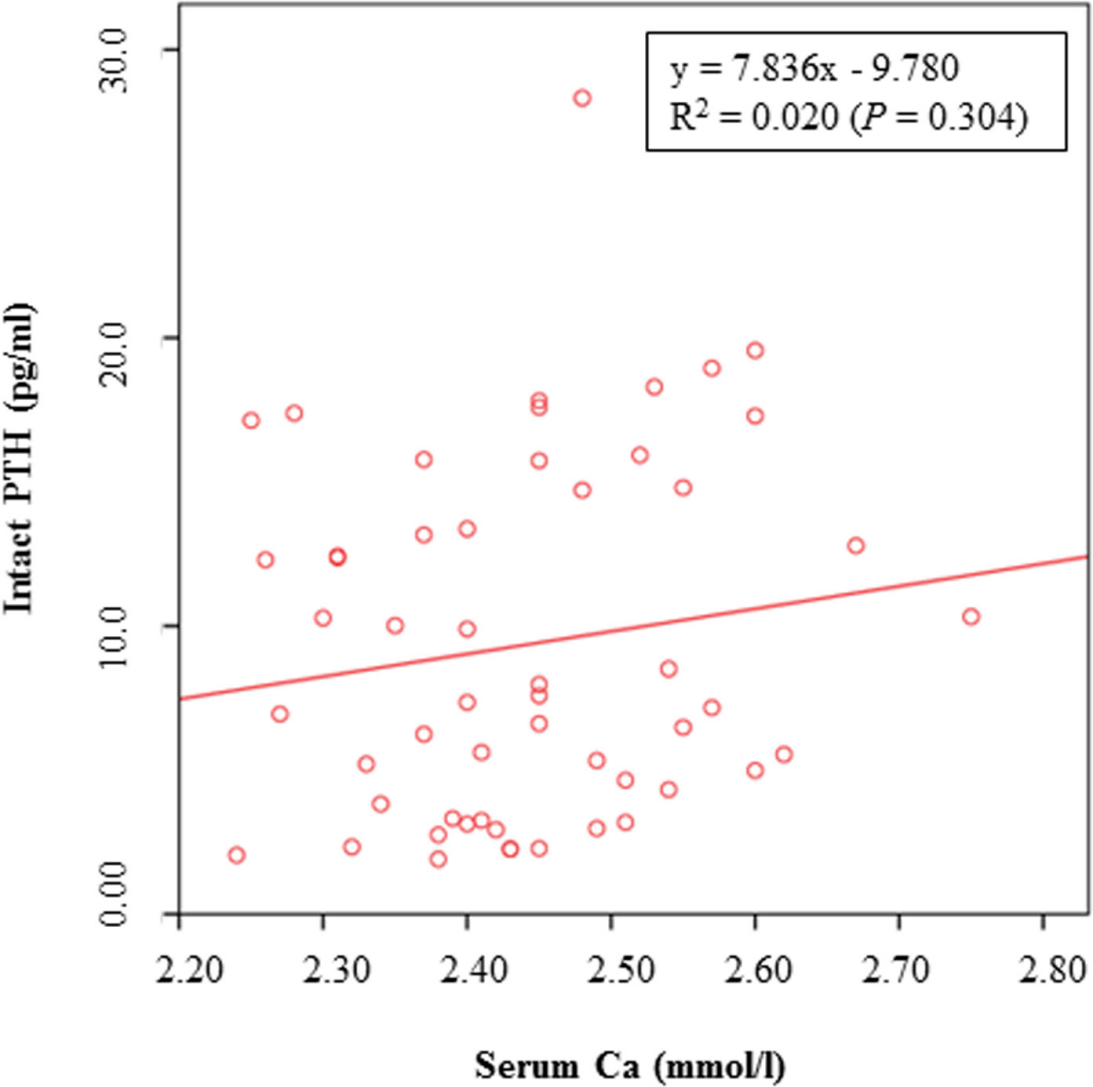
Concentrations of iPTH as a function of Ca in the serum of cats fed a diet with different levels of dicalcium phosphate.

**Fig 8:**
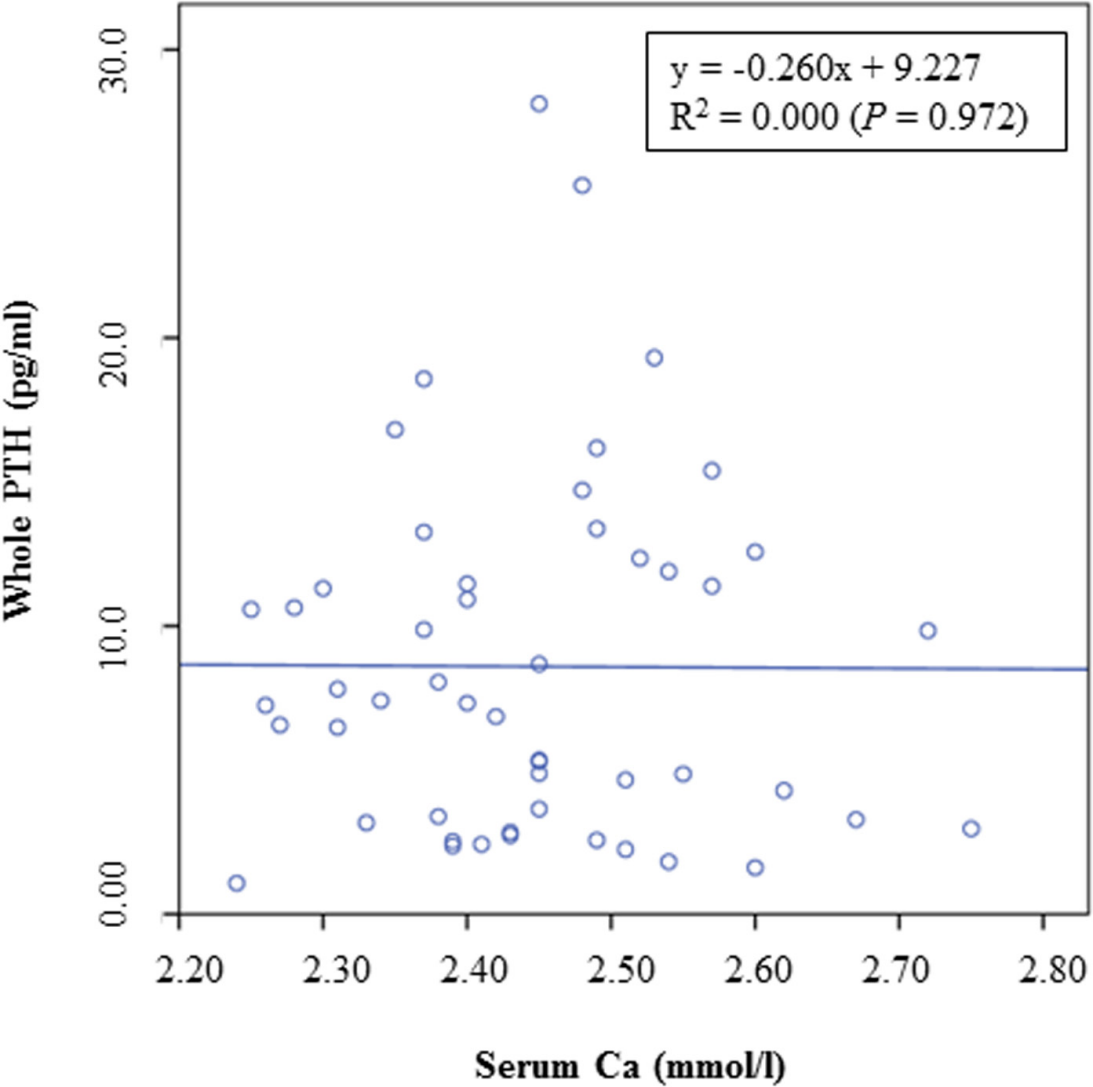
Concentrations of wPTH as a function of Ca in the serum of cats fed a diet with different levels of dicalcium phosphate.

The results of the present study could not demonstrate a decrease of PTH with increasing levels of dietary Ca, as previously described [23]. This difference between the previous and the present study might be explained by the different sources of dietary Ca or by the observed individual variations in the serum PTH concentrations of the cats in the present study. It is also interesting to notice that iPTH concentrations were partly lower when compared to wPTH concentrations (Fig 5). When iPTH was graphed as a function of wPTH (Fig 6), a significant correlation between both parameters was observed, although the coefficient of correlation was only small. PTH is a polypeptide consisting of 84 amino acids [31]. According to the manufacturer of the PTH assay (Scantibodies Laboratory, Inc., Santee, CA, USA), measurement of iPTH includes the detection of the bioactive wPTH (1–84) and N-truncated PTH fragments. Therefore, higher iPTH concentrations than wPTH concentrations would be expected. However, a previous study by Pineda et al. [32] also demonstrated greater wPTH concentrations than iPTH concentrations in the plasma of cats. The authors used the same immunoradiometric assay as considered for the present study and suggested a higher affinity of the tracer antibody against the N-terminal PTH region (wPTH) when compared to the affinity of the iPTH tracer [32]. However, in this study, both, the iPTH and wPTH assays produced valid results in hypo- and hypercalcemic cats, and the authors concluded that these assays can therefore be used to measure PTH in cat blood [32]. Moreover, PTH secretion in hypocalcemic state was observed to be a sigmoidal curve, whereas consistent PTH inhibition was detected in hypercalcemic cats [32]. In the present study, cats were normocalcemic and no impact on PTH secretion could be observed (Figs 7 and 8).

The unaffected serum Ca and phosphate concentrations might also explain why FGF23 serum concentrations remained unchanged by varying dietary Ca concentrations. It has been demonstrated that hypercalcemia [6] and hyperphosphatemia [3] increase plasma FGF23 concentrations in mice and cats. Hypocalcemia was associated with decreased plasma FGF23 concentrations in rats [5].

For the interpretation of the present data concerning the concentrations of PTH, FGF23 and the metabolites of vitamin D in the blood of the cats, it should also be considered that the experimental diets did not only differ in Ca, but also in P levels. We deliberately decided to use dicalcium phosphate in order to maintain a constant Ca:P-ratio in the experimental diets. Recommendations suggest that the Ca:P-ratio for cat food should be between 0.9–1.1:1 [33], and it has also been suggested that ratios below 1 could negatively affect renal function [34]. In addition, it has been demonstrated that a wide Ca:P-ratio decreases the absorption of P in the intestine [34]. In contrast, no effect on the intestinal Ca absorption was observed when the P levels in the diet were increased and the Ca level remained unchanged [22]. In order to ensure that the intestinal absorption of Ca or P would not be negatively affected by the Ca:P-ratio, the present study design thus provided experimental diets which were close to the recommended ratio. However, the increasing P levels in the diets should be considered with regard to the PTH, FGF23 and calcitriol secretion. Increased phosphate concentrations in the serum lead to a reduced secretion of calcitriol [35], resulting in a reduced turnover of Ca and phosphate from bones and a lower absorption of Ca and phosphate in the intestine. As a result, blood Ca concentrations decrease and the secretion of PTH increases. The high PTH levels enhance the renal P excretion [36, 37], which leads to a reduction of the P concentrations in the blood. Moreover, hyperphosphatemia also stimulates FGF23 secretion, resulting in an increased renal P excretion, a reduced intestinal P absorption and decreased plasma PTH and calcitriol concentrations [2]. In the present study, cats were normophosphatemic and serum FGF23 levels were only numerically increased with the high Ca diet. However, the described mechanisms underline that both the increased dietary Ca and P levels could be related to the observed decrease of the precursors of calcitriol (25(OH)D_2_ and 25(OH)D_3_) in the serum of the cats.

The present results further demonstrated that P excretion of the cats was regulated to a large extent by adjusting urinary excretion. This relationship was not observed in our previous study [23], where high P levels from bone meal in a canned diet only led to increased faecal P concentrations, while the urinary P concentrations remained unchanged. These divergent results might be explained by the markedly higher dietary P levels in the present study, but also indicate a different availability of minerals from bone meal compared to dicalcium phosphate.

The divergence between urinary P concentration and urinary Ca concentration in the current study is probably ultimately a direct result of the uncoupling between the intestinal absorption of the two ions and might be an explanation for the marked effects of the diets on the urinary pH of the cats. We demonstrated a significant decrease of the urinary pH (fasting/postprandial) from 7.09/6.91 (0.6% Ca) to 6.02/6.01 (2.2% Ca). Such acidotic effects are to be expected if a strong anion such as phosphate is absorbed from the intestine unaccompanied by a strong cation. In this situation, protons will follow the strong anion (here: phosphate) and have to be renally excreted, explaining the drop in urinary pH observed. Notably, acidosis can be expected to enhance PTH secretion [38]. This may have compensated for any decrease in PTH secretion caused by the increase in dietary Ca, so that in conjunction, no significant dietary impact on PTH secretion could be observed in this study.

The small increase of the urinary pH after feeding the diet 2.4% Ca (6.22/6.19) may be explained by the reduction of the feed intake of the cats in this period, resulting in a reduced mineral intake. Although the differences in the feed intake were not statistically significant, they may be relevant for the interpretation of the results on urinary pH, since urinary P concentrations did not markedly increase after feeding the diet 2.4% Ca when compared to the diet 2.2% Ca.

The measured pH values are of practical importance for the aetiology and prevention of feline uroliths, but also with regard to acid base balance. A low urinary pH, particularly a value < 6.29, is considered to prevent magnesium ammonium phosphate (MAP) uroliths, but to enhance the risk for the development of calcium oxalate (CaOx) stones [39, 40]. Thus, feeding diets with higher levels of dicalcium phosphate (1.9–2.4% Ca) could be critical for the prevention of CaOx crystals and stones because of the demonstrated low urinary pH. On the other hand, MAP can efficiently crystallize at pH values > 7 [20]. The diet with the lowest concentration of dicalcium phosphate (0.6% Ca) would therefore enhance the risk for the formation of MAP stones, whereas the measured urinary pH values after feeding the diets 0.8% Ca and 1.5% Ca are neither critical for the formation of MAP nor CaOx uroliths.

Besides the impact on urolith crystallisation, the urinary pH should also be discussed with regard to acid base balance in cats. We observed an acidotic effect of high levels of dicalcium phosphate in the diets on urinary pH, potentially induced by an enhanced renal P excretion. Acidification of the organism in terms of development of metabolic acidosis can negatively affect several functions of the organism, for instance cardiovascular function, glucose metabolism or bone turnover [41]. In the present study, pH and bicarbonate concentrations in the blood of the cats have not been measured, why potential acid-base-disorders could not be conclusively identified. However, the low urinary pH (6.02/6.01) when feeding the diet 2.2% Ca revealed a strong dietary acidifying effect. Long-term studies should evaluate the physiological relevance of chronic acidification by high dietary dicalcium phosphate concentrations. In this context, it should be considered that dietary Ca sources differ in commercial diets for cats [1]. In order to maintain an adequate Ca:P-ratio, high Ca diets might also be high in P. It is interesting to notice that the urinary pH was not affected by increasing levels of bone meal in a canned diet for cats [23]. Since the urinary P concentrations were unaffected in this previous study while faecal P rose with dietary intake, it can be hypothesized that P was more efficiently absorbed in the present study, leading to the strong decrease in urinary pH. It can therefore be concluded that not only the dietary Ca and P level, but also the Ca and P source in a diet is of high physiological significance.

With regard to the urinary pH, it should finally not go unmentioned that a low urine pH was not associated with an increase in renal Ca excretion. In human beings, an acid load, derived from a high intake of animal protein or especially sulphur-containing amino acids, has been discussed to increase renal Ca excretion [42, 43]. On the other hand, other authors also found an enhanced renal Ca excretion when alkalizing components were added to a high-protein diet [44, 45], indicating that not only an acid load leads to an increase of urinary Ca in humans. Based on the present findings, urinary Ca excretion in cats seems to be widely independent of dietary factors, since no effect of dietary Ca levels or a diet-induced acidic urine could be observed. The faecal Ca concentrations increased with higher Ca levels in the diets, indicating that regulatory mechanisms may have limited the uptake of Ca by the intestinal tract. A recent meta-analysis also demonstrated an increase of faecal Ca excretion in cats with increasing dietary Ca concentrations [46].

Higher intestinal Ca concentrations could be interesting with regard to CaOx urolith prevention. Recent studies from humans and dogs indicate that high dietary Ca concentrations could be advantageous [47, 48], because of an intestinal complexation between Ca and Ox, lowering their intestinal absorption and renal excretion and therefore reducing the risk for the development of CaOx or other Ca containing crystals and stones. In the present study, urinary Ox concentrations were comparable when the diets with the low and moderate Ca levels (0.6–1.5% Ca) were fed, but decreased when feeding the high-Ca diets (1.9–2.4% Ca). Thus, it can be assumed that the hypothesis of an intestinal complexation between Ca and Ox in the case of high Ca levels in a diet [47, 48] could also apply to cats. However, it should be considered that the amounts of Ox are normally low in cat food [49], limiting its complexation with Ca in the intestine. Moreover, the demonstrated decrease of the urinary Ox concentrations with increasing dietary Ca levels was only moderate, indicating only a small risk reduction for the formation of feline CaOx uroliths by higher dietary Ca levels.

## Conclusion

The present study demonstrated that the Ca excretion of cats is mainly regulated by changes in intestinal absorption and not via adjustment of renal excretion. Although the PTH and FGF23 concentrations in the blood of the cats were unaffected by increasing levels of dietary Ca, the observed decrease of the calcitriol precursors 25(OH)D_2_ and 25(OH)D_3_ indicates diet-dependent hormonal control mechanisms. High dietary dicalcium phosphate levels were associated with a low urine pH. Future studies should investigate the physiological relevance of the acidifying effect in the feline organism.
